# Genetic analysis of admixture and hybrid patterns of *Populus hopeiensis* and *P*. *tomentosa*

**DOI:** 10.1038/s41598-019-41320-z

**Published:** 2019-03-18

**Authors:** Dongsheng Wang, Zhaoshan Wang, Xiangyang Kang, Jianguo Zhang

**Affiliations:** 1 State Key Laboratory of Tree Genetics and Breeding, Key Laboratory of Silviculture of the State Forestry Administration, Research Institute of Forestry, Chinese Academy of Forestry, Beijing, 100091 China; 2 Collaborative Innovation Center of Sustainable Forestry in Southern China, Nanjing Forestry University, Nanjing, 210037 China; 3 College of Horticulture Sciences & Technology, Hebei Normal University of Science & Technology, 066004 Qinhuangdao, China; 4 College of Biological Sciences and Biotechnology, Beijing Forestry University, Beijing, 100091 China

## Abstract

Hybridization and introgression have resulted in reticulate evolution within the genus *Populus*. Consequently, the origin and evolutionary history of some hybrids has become blurred. *P*. *hopeiensis* and *P*. *tomentosa* are endemic to China, and there is still controversy about their origin. We employ phylogeny, Bayesian estimation of admixture, and approximate Bayesian computation to investigate their origin with 10 nuclear DNA and 6 cpDNA regions. The combined evidences firmly support the hypothesis that they are hybrids and dominated by F_1_s. *P*. *hopeiensis* was generated via hybridization between the paternal species *P*. *alba* and maternal species *P*. *davidiana*. Surprisingly, *P*. *tomentosa* was divided into two genetic types with different maternal parents. *P*. *adenopoda* hybridized with *P*. *alba* directly to generate the first genetic type (mb1) and hybridized with *P*. *davidiana* followed by *P*. *alba* to generate the second (mb2). In both genetic types, *P*. *alba* acted as the male parent. The maternal parent was *P*. *adenopoda* and *P*. *davidiana* for mb1 and mb2, respectively. Hybridization not only generated these hybrids but also resulted in a unidirectional gene flow from *P*. *davidiana* to *P*. *adenopoda*. The *Populus* species have maintained a delicate balance between their genetic integrity and gene exchange.

## Introduction

It has long been known that hybridization and introgression play key roles in evolution^1,2^. Natural interspecific hybridization has been estimated to be present in approximately 25% of all plant species^3^. Hybridization can trigger speciation^4^ but can also blur species boundaries and complicate the evolutionary history of related taxa^5^. Many taxonomic debates regarding the origins of various taxa have resulted from the confusing artifacts of hybridization and followed introgression^6–8^. Introgression, the movement and subsequent stable incorporation of genes from one species to the gene pool of another, is caused by hybridization and repeated backcrossing^9^. Thus, introgression is a special conduit for gene flow^10^. The spatial scale and geographic pattern of introgression are influenced by a variety of factors, including natural selection, individual dispersion distance and the place where the hybridization took place^11^.

Hybrid zones provide a window for the study of hybrid production and interspecific gene flow. A typical hybrid zone is a narrow region where different species meet, exchange genes, and produce hybrids^12^. Many hybrids and introgressive hybridization in *Populus* are found in these typical hybrid zones^13,14^. However, some hybrids jump out of the typical hybrid zone, appearing a few hundred kilometers from the nearest parent^15^. The geographical isolation creates conditions for production of hybrids, but also makes it difficult to distinguish them and verify their parentage. Cytonuclear discordance, which is a topological incongruity between the maternally inherited cpDNA (chloroplast DNA) and biparentally inherited nuclear DNA^16,17^, has received much attention within hybrid zones. Cytonuclear discordance is a useful tool to decipher the direction of introgression and whether certain species are the parental contributors to hybrids^18^.

Hybridization and introgression also appear to have played an essential role in diversification of *Populus* L. given that many members were involved in ancient hybridization events^15,19,20^. Species of section *Leuce* Duby tend not to hybridize with the other five sections of *Populus* (*Abaso*, *Aigeiros*, *Leucoides*, *Tacamahaca*, and *Turanga*), but exhibit widespread intrasectional hybridization and introgression^19,21^. Section *Leuce* itself is even thought to originate from hybridization between members of sections *Leucoides* and *Turanga*^22^. As such, the number of species in section *Leuce* remains under debate, varying from 8 to 10^15,23,24^. Some species are sympatric, providing many opportunities for hybridization and introgression and following some natural hybrids and/or interspecific gene flow^25^. Some natural hybrids, such as *P*. *canescent* (Ait.) Smith., survive by asexual reproduction^26,27^. The origins and evolutionary histories of some hybrid poplars have been studied extensively^28,29^, but are still ambiguous for some native poplars in China.

Both *P*. *hopeiensis* Hu & H. F. Chow and *P*. *tomentosa* Carr. are native to China with high quality timber^24,30,31^. Indeed, the origins of *P*. *hopeiensis* and *P*. *tomentosa* have been subject to much debate. Based on morphological characteristics, it is hypothesized that *P*. *tomentosa* and *P*. *davidiana* Dode are the parent species of *P*. *hopeiensis*^24^, but this hypothesis is rejected after reciprocal cross experimentation^32^. However, *P*. *hopeiensis* is suspected to have a close relationship with *P*. *davidiana*^33^. Despite the potential value of *P*. *hopeiensis* for the evolutionary analysis of hybridization, the genetic documentation of its putative parents is lacking.

The origin of *P*. *tomentosa* is more ambiguous; even the number of its parental species is debated. Although several workers have suggested that *P*. *tomentosa* originated from the hybridization of two taxa, varying parental species have been proposed. Bartkowiak concludes that *P*. *tomentosa* is derived from *P*. *alba* L. × *P*. *tremula* L. (♀ × ♂) based on the characters of parviflorous bracts^34^. In contrast, *P*. *alba* and *P*. *davidiana* are believed to act as the parents of *P*. *tomentosa* with morphological and anatomical characters^35^. RAPD (random amplified of polymorphic DNA) analysis suggests that *P*. *tomentosa* originated from *P*. *alba* (♀) and *P*. *adenopoda* Maxim. (♂)^36^. However, phylogeny of the *Populus* genus based on DNA sequences indicates that *P*. *tomentosa* is derived from *P*. *davidiana* (♀) and *P*. *adenopoda* (♂)^33^. In contrast, other researchers have suggested that *P*. *tomentosa* has more than two parental species^37,38^. Isoenzyme analysis indicates that three species, *P*. *alba*, *P*. *adenopoda* and *P*. *davidiana*, formed *P*. *tomentosa*, but the author unfortunately did not identify the maternal or paternal parent^37^. Although many studies have proposed that *P*. *tomentosa* is a natural, spontaneous hybrid, no solid evidence is available to verify its parents.

Numerous investigations have sought to clarify the origin of *P*. *hopeiensis* and *P*. *tomentosa*. Unfortunately, a dearth of studies based on gene sequence impedes the identification of their origin. Previous studies have been hampered partly by limited sampling. Indeed, nearly all the Eurasian species in section *Leuce* (*P*. *alba*, *P*. *tremula*, *P*. *adenopoda*, and *P*. *davidiana*) have been proposed as parent species of *P*. *hopeiensis* or *P*. *tomentosa*. In addition, close relationships occur among *P*. *tremula*, *P*. *tremuloides* Michaux, and *P*. *grandidentata* Michx^15,39^, making characterizing parentage challenging. Therefore, to clarify their origins, we used 392 individuals in 36 populations of 8 taxa with 10 nuclear DNA and 6 cpDNA sequences. More generally, we aimed to improve our understanding of hybridization and introgression in section *Leuce* and to lay the foundation for the conservation of genetic resources and breeding innovation.

## Results

### Polymorphic analyses and neutral test

The length of the aligned nuclear DNA sequences ranged from 388 to 808 bp, and the concatenated length of all ten nuclear loci was 5715 bp (Table S1). The aligned cpDNA sequences ranged from 945 to 2551 bp, and the concatenated length of all six cpDNA loci was 7741 bp (Table S1).

DnaSP v5^40^ was used to analyze polymorphic and test neutrality of variation. All taxa had generally high nucleotide diversity in the nuclear loci (Table S2), ranging from 0.00281 (*P*. *grandidentata*) to 0.00802 (*P*. *adenopoda*). The overall nucleotide diversity of cpDNA was lower than that of nuclear DNA, ranging from 0.0001 (*P*. *grandidentata*) to 0.00103 (*P*. *davidiana*).

Some of the nuclear loci significantly departed from neutrality based on Tajima’s *D*, Fu and Li’s D* and Fu and Li’s F*, such as locus 6 in *P*. *tremuloides* and locus YLT24 in *P*. *tremula* (Table S2). Conversely, our MLHKA analysis only supported a significant difference between the neutral model and the selection model at locus 6 of *P*. *tremuloides* (Table S3).

### Phylogenetic analyses

We firstly recovered ten individual nuclear phylogenetic trees of section *Leuce*. In these individual gene trees, it was found that most of the *P*. *hopeiensis* were clustered together with *P*. *alba*, *P*. *davidiana* and *P*. *tomentosa*, whilst the most *P*. *tomentosa* were clustered together with *P*. *alba*, *P*. *davidiana*, *P*. *adenopoda* and *P*. *hopeiensis*. Some *P*. *adenopoda* were clustered together with *P*. *davidiana* (Fig. S1).

**Figure 1:**
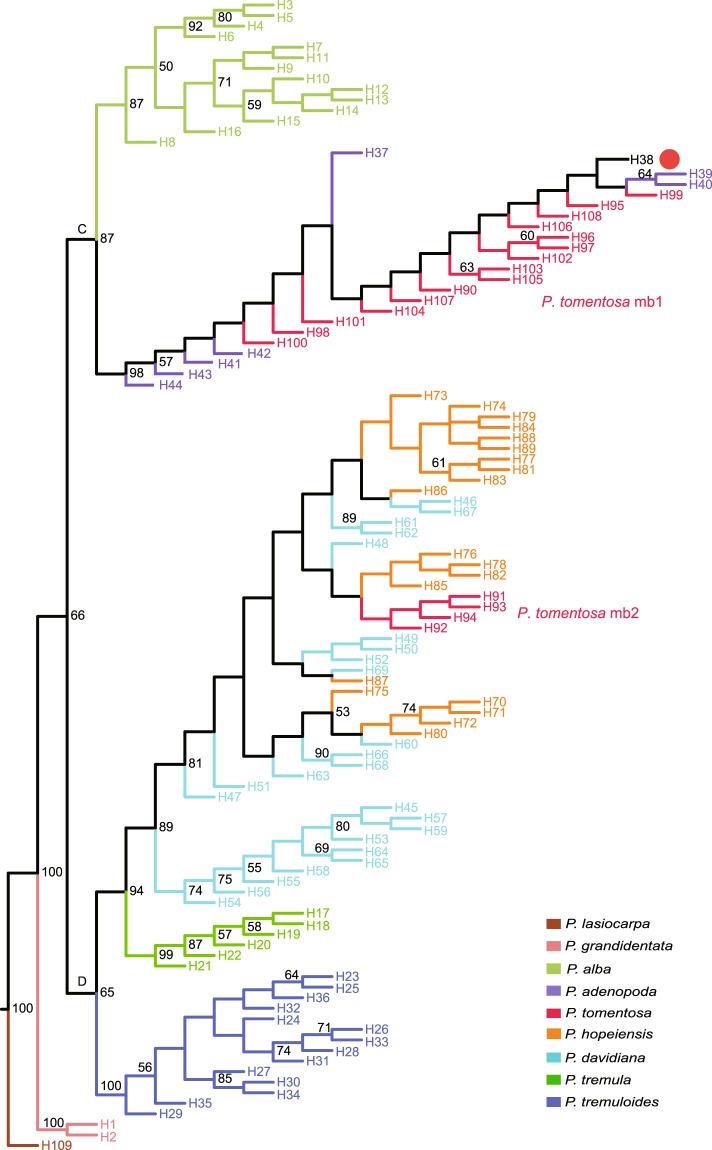
Maximum parsimony (MP) phylogeny of *Populus* section *Leuce* based on an analysis of six concatenated cpDNA regions. Only bootstrap (BS) values > 50% are shown. Different colors indicate different taxa. Leaf nodes show the haplotype numbers. The pie chart indicates that H38 was found in 1 individual of *P*. *adenopoda* and 191 individuals of *P*. *tomentosa* mb1.

Phylogenies of both concatenated nuclear DNA and cpDNA datasets were well resolved (Figs 1 and S2). Section *Leuce* formed a highly supported (Posterior probability, PP = 1) clade with *P*. *grandidentata* sister to the remaining taxa in the concatenated nuclear phylogenetic tree. The others fell into two major clades (clade A and B). Within clade A, some *P*. *tomentosa* sequences grouped with *P*. *adenopoda* in clade A1 with high support (PP = 1) and these were sister to clade A2. The other *P*. *tomentosa* sequences, *P*. *hopeiensis* and *P*. *alba*, formed the well supported clade A2 (PP = 0.96). Although *P*. *tomentosa* was located in different clades, for any sample of *P*. *tomentosa*, one of its two sequences is located in clade A1. The other sequence is located in clade A2. Clade B was composed of *P*. *davidiana*, *P*. *tremula*, *P*. *tremuloides* and a few *P*. *adenopoda* sequences that were lowly resolved. Although sequences from the same species did not always group together, clusters formed by sequences from different species were poorly supported (Fig. S2).

Three cpDNA trees also had approximately the total same topology (Figs 1, S3 and S4). The positions of eight taxa were identical across trees with minor differences only noted in the terminal positions of some haplotypes within a taxon. Similar to the nuclear DNA phylogeny, section *Leuce* was monophyletic with high support across all cpDNA phylogenetic analyses (Bootstrap, BS = 100, Fig. 1). The analysis also revealed that *P*. *grandidentata* diverged first with clade C and clade D, forming a sister relationship. The moderately supported group C (BS = 87) was composed of *P*. *adenopoda*, *P*. *alba*, and the major haplotypes of *P*. *tomentosa* (named by genetic types *P*. *tomentosa* mb1; Table 1, Fig. 1). *P*. *tomentosa* mb1 (15 haplotypes, 217 individuals) first grouped with *P*. *adenopoda*, and they were sister to *P*. *alba* (BS = 87). Clade D was also recovered with weak support (BS = 65) and contained *P*. *hopeiensis*, *P*. *davidiana*, *P*. *tremula*, *P*. *tremuloides*, and the remainder of the haplotypes of *P*. *tomentosa* (*P*. *tomentosa* mb2; Fig. 1). *P*. *tremuloides* was sister to others in this group. *P*. *tomentosa* mb2 (4 haplotypes, 18 individuals) clustered with *P*. *hopeiensis* and *P*. *davidiana* (BS = 89) with *P*. *tremula* sister to these with good support (BS = 94).

**Table 1:** The number of specimens collected per locality of the two *Populus  tomentosa* genetic types.

Types	Origins							Total
	MBJ	MHBJ	MHN	SXJ	MSXQ	MGS	AH	
*P*. *tomentosa* mb1	28	46	55	42	34	6	6	217
*P*. *tomentosa* mb2	9	7	0	1	1	0	0	18
Total	37	53	55	43	35	6	6	234

Note: Collection localities are detailed in Tables S4 and S5. All localities are in China: MBJ, Beijing; MHBJ, Hebei; MHN, Henan; SXJ, Shanxi; MSXQ, Shaanxi; MGS, Gansu; AH, Anhui.

### Genetic structure analyses based on ten nuclear DNA

**Figure 2:**
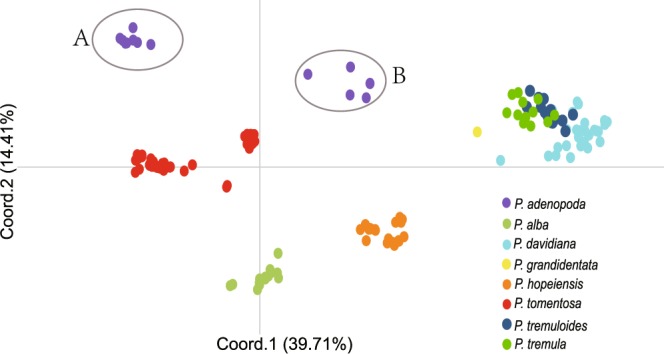
Principal component analyses (PCA) for 10 nuclear DNA based on genetic distance. Different taxa are represented with different colors. Each circle represents an individual. The x-axis and the y-axis represent principal components 1 and 2, respectively. The percentage in parentheses represents the observed variance.

Clustering analysis for nuclear DNA was conducted using principal component analysis (PCA) in GENALEX 6.5 to detect complex patterns of genetic structure^41^. The PCA based on genetic distance demonstrated that *P*. *hopeiensis* was located between *P*. *davidiana* and *P*. *alba* with closer affinity with the later. Whilst *P*. *tomentosa* was located among *P*. *adenopoda*, *P*. *alba* and *P*. *davidiana*, with a closer affinity with the first two. It is worth noting that *P*. *adenopoda* is divided into two clusters (A and B), cluster B is located between cluster A and *P*. *davidiana*. Cluster B is composed of those individuals which appear to have undergone introgression with *P*. *davidiana* (discussed below; Fig. 2).

**Figure 3:**
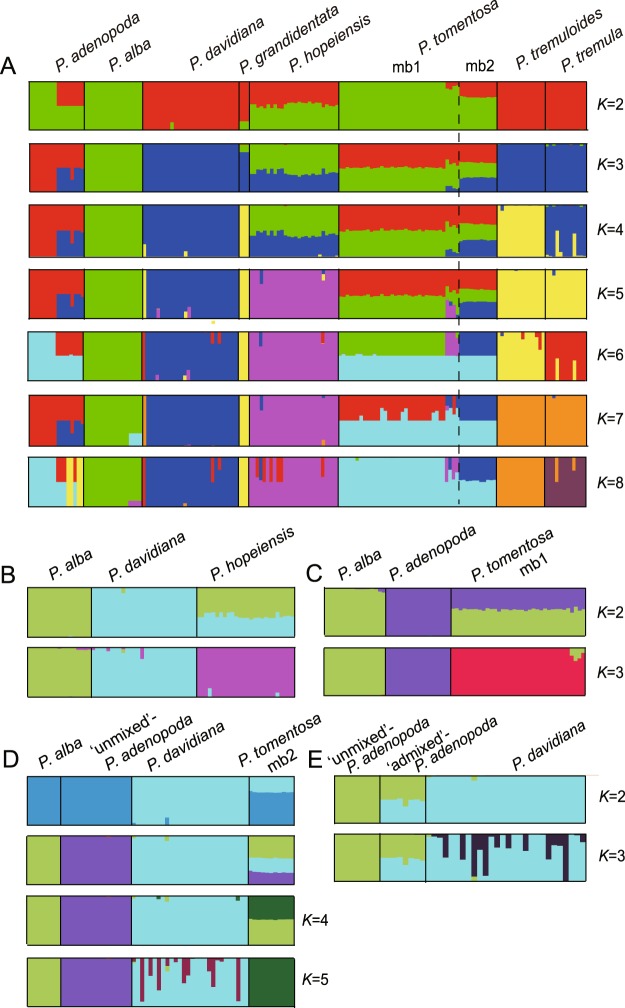
STRUCTURE results based on ten nuclear DNA dataset. Taxa and subgroups are shown along the x-axis, and values of *K* are shown on the y-axis. (**A**) STRUCTURE results for all taxa analyzed in this study with usepopinfo = 1; note that the optimum number of clusters was 3. (**B**) Results of the STRUCTURE analysis of *P*. *hopeiensis* and its putative species parents (*P*. *alba* and *P*. *davidiana*) only. (**C**) Results of the STRUCTURE analysis of *P*. *tomentosa* mb1 and its putative species parents (*P*. *alba*, and *P*. *adenopoda*) only. (**D**) Results of the STRUCTURE analysis of *P*. *tomentosa* mb2 and its putative parents (*P*. *alba*, *P*. *davidiana*, and *P*. *adenopoda* (‘unmixed’)) only. (**E**) Results of the STRUCTURE analysis of *P*. *adenopoda* and *P*. *davidiana*; *P*. *adenopoda* is separated into two groups where evidence of introgression by *P*. *davidiana* was detected (‘admixed’) and where no evidence of introgression was detected (‘unmixed’) based on A.

To assign the individuals to the populations and estimate potential admixture, STRUCTURE^42^ was used to estimate the overall genetic structure of all taxa with 10 nuclear loci. STRUCTURE analysis indicated that all populations likely fell into three genetic clusters at the optimal number of modelled populations (*K* = 3, usepopinfo = 1) (Fig. S5A,B). At *K* = 3, *P*. *adenopoda* fell into one cluster with four individuals (three from Hubei and one from Guizhou) mixed by other species. *P*. *alba* fell into another cluster, and *P*. *davidiana*, *P*. *grandidentata*, *P*. *tremuloides* and *P*. *tremula* fell into the third cluster (Fig. 3A). *P*. *hopeiensis* were a mixture of two clusters (Fig. 3A). *P*. *tomentosa* could be split into two genetic types (*P*. *tomentosa* mb1 and mb2) along the division recovered in the cpDNA phylogenies. Most individuals of *P*. *tomentosa* mb1 were a mixture of *P*. *adenopoda* and *P*. *alba*, and *P*. *tomentosa* mb2 was a mixture of all three clusters (Fig. 3A). It is notable that four individuals assigned to *P*. *tomentosa* mb1 based on our phylogenetic analysis (three individuals from Shaanxi and one from Hebei) were shown to be a mixture of all three clusters (Fig. 3A). Although the genetic structure of *P*. *hopeiensis* and *P*. *tomentosa* were unclear at *K* = 3, genetic affinities were clearer at higher values of *K* combined with phylogeny results. The clustering patterns at *K* = 4 and *K* = 5 indicated that *P*. *alba* and *P*. *davidiana* contributed genetic material to *P*. *hopeiensis*, whereas *P*. *davidiana* contributed to *P*. *tomentosa* mb2 and some individuals of *P*. *tomentosa* mb1. Finally, the mosaic individuals of *P*. *adenopoda* were admixed with *P*. *davidiana* (named admixed *P*. *adenopoda* for convenience). The most likely number of clusters was *k* = *2* when the STRUCTURE analysis was conducted without location information (usepopinfo = 0) (Fig. S5C,D). The cluster results generated without location information were very similar to those generated with location information (Fig. S6).

To further investigate these hypothesized clusters, we performed four hierarchical analyses. We first analyzed *P*. *hopeiensis*, *P*. *alba*, and *P*. *davidiana* alone. Our results indicated that the most likely number of clusters was *K* = 2 (Fig. S5E,F). In addition, *P*. *alba* and *P*. *davidiana* clustered in distinct groups with high probability (Fig. 3B). *P*. *hopeiensis* was admixed and assigned to both clusters with moderate probability. *P*. *hopeiensis* was assigned to a discrete cluster at *K* = 3 (Fig. 3B). We next analyzed *P*. *tomentosa* mb1, *P*. *alba*, and *P*. *adenopoda* alone. In this analysis, we found that *P*. *tomentosa* mb1 was a mixture of *P*. *alba* and *P*. *adenopoda* at optimal cluster *K* = 2 but was recovered as a distinct cluster at *K* = 3 (Figs 3C and S5G,H). To investigate the precise genetic composition of *P*. *tomentosa* mb2, we analyzed *P*. *alba*, *P*. *davidiana*, and ‘unmixed’ *P*. *adenopoda*. At optimal cluster *K* = 3, *P*. *alba*, *P*. *davidiana* and ‘unmixed’ *P*. *adenopoda* were recovered as separate clusters, whereas *P*. *tomentosa* mb2 was a mixture of all three (Figs 3D and S5I,J). At *K* = 4, *P*. *tomentosa* mb2 was mixed with 2 clusters, including *P*. *alba*. At *K* = 5, *P*. *tomentosa* mb2 was recovered as a distinct cluster (Figs 3D and S5I,J). Finally, we analyzed *P*. *adenopoda* and *P*. *davidiana* alone. We found that at *K* = 2 (Fig. S5L,M), some individuals of *P*. *adenopoda* showed mixing from *P*. *davidiana*, and these individuals did not form a distinct cluster at *K* = 3(Fig. 3E).

### Classification analysis

**Figure 4:**
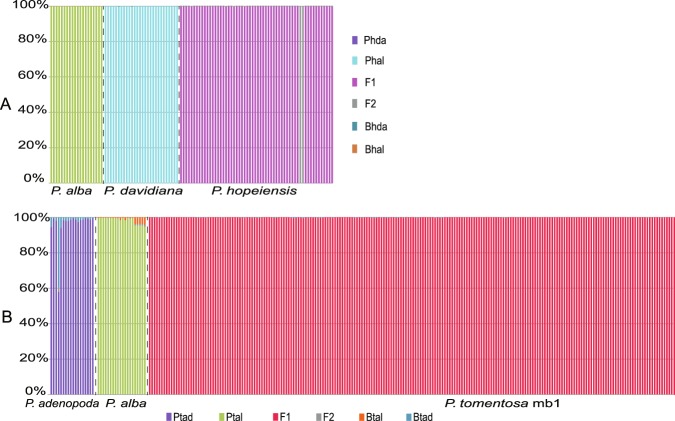
Results of Newhybrids for hybrids and their parents. Each bar represents an individual, and different colors indicate different genetic classes. Subfigure (**A**), analysis of *P*. *hopeiensis* and its putative parents. Phal and Phda are short for pure parent *P*. *alba* and pure parent *P*. *davidiana*, respectively. Bhal and Bhda represents for the genotype generated by F_1_ backcrossing with *P*. *alba* and with *P*. *davidiana*, respectively. Subfigure (**B**), analysis of *P*. *tomentosa* mb1 and its putative parents. Ptal and Ptad are short for pure parent *P*. *alba* and *P*. *adenopoda*, Btal and Btad represents for the genotype generated by F_1_ backcrossing with *P*. *alba* and *P*. *adenopoda* respectively. Y-axes represents the posterior probability of each sample assigned to a genotype class.

Based on the genotype posterior probability, Newhybrids v1.1^43^ was used to identify and characterize the hybrids in the admixed populations. Newhybrids analysis indicated that all samples could be confidently assigned to a particular genotype class. This analysis recovered all specimens of *P*. *alba* and *P*. *davidiana* as pure parents with high support (posterior probability (PP) >99; Fig. 4A). Most specimens of *P*. *hopeiensis* were classified as F_1_s; only two individuals were classified as F_2_s (Fig. 4A). Similar results were obtained when analyzing the putative parents of *P*. *tomentosa* mb1 (Fig. 4B). All specimens of *P*. *alba* and *P*. *adenopoda* were recovered as pure parents, whereas all specimens of *P*. *tomentosa* mb1 were classified as F_1_s. This finding also indicated the hybrid origin of *P*. *hopeiensis* and *P*. *tomentosa* mb1.

### Demographic estimates using Bayesian approximation

**Figure 5:**
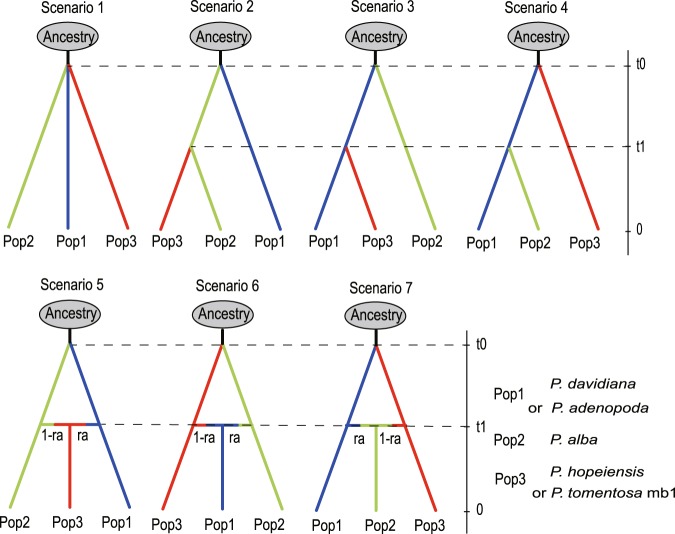
Seven evolutionary scenarios of *P*. *hopeiensis* and *P*. *tomentosa* mb1 tested by DIYABC. For *P*. *hopeiensis*, Pop1, Pop2, Pop3 represents *P*. *davidiana*, *P*. *alba* and *P*. *hopeiensis* respectively. For *P*. *tomentosa* mb1, Pop1, Pop2, Pop3 represents *P*. *adenopoda*, *P*. *alba*, and *P*. *tomentosa* mb1 respectively. t# is on behalf of time that scale measured in generations, N# effective population size of each populations (Pop 1, 2, 3, a) in the corresponding period, r# admixture rates.

**Figure 6:**
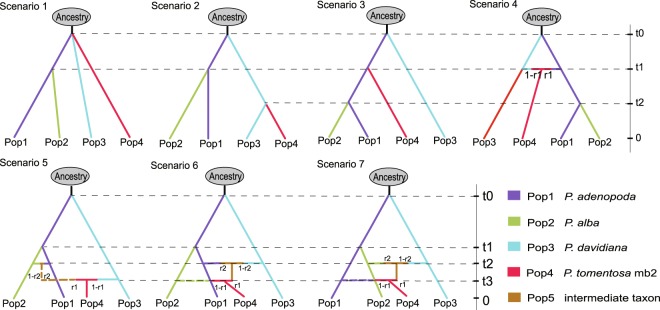
Seven evolutionary scenarios of *P*. *tomentosa* mb2 tested by DIYABC. t# represents time that scale measured in generations, N# effective population size of each populations (Pop 1, 2, 3, a) in the corresponding period, r# admixture rates. Pop1, Pop2, Pop3, Pop4, and Popa were on behalf of *P*. *adenopoda*, *P*. *alba*, *P*. *davidiana*, *P*. *tomentosa* mb2 and ancestral population, respectively. Pop5 was the intermediate taxon that derived from the hybridization between two taxa of Pop1, Pop2 and Pop3.

To further investigate the origin pattern of *P*. *hopeiensis* and *P*. *tomentosa* (mb1 and mb2), seven alternative scenarios (Figs 5 and 6) were summarized and tested with DIYABC V 2.1.0^44^. In the analysis of *P*. *hopeiensis*, scenario 5 exhibited the highest support (PP = 0.9700; 95% confidence interval: 0.9445–0.9912; Table 2), suggesting that *P*. *alba* and *P*. *davidiana* hybridized and generated *P*. *hopeiensis* (Fig. 5).

**Table 2:** Posterior probabilities with 95% confidence intervals of scenarios for *P*. *hopeiensis* and *P*. *tomentosa*.

Taxa	Scenarios	Posterior probability	95% CIs
*P*. *hopeiensis*	1	0.0019	[0.0023–0.1035]
	2	0.0016	[0.0003–0.1029]
	3	0.0258	[0.0061–0.1367]
	4	0.0002	[0.0000–0.1006]
	5	**0**.**9700**	[0.9445–0.9912]
	6	0.0004	[0.0001–0.1011]
	7	0.0001	[0.0000–0.1005]
*P*. *tomentosa* mb1	1	0.0001	[0.0000–0.6691]
	2	0.0000	[0.0000–0.6691]
	3	0.0219	[0.0000–0.6878]
	4	0.0000	[0.0000–0.6690]
	5	**0**.**9780**	[0.9626–0.9923]
	6	0.0000	[0.0000–0.6691]
	7	0.0000	[0.0000–0.6690]
*P*. *tomentosa* mb2	1	0.0016	[0.0000–0.9307]
	2	0.0006	[0.0000–0.9527]
	3	0.0020	[0.0000–0.9297]
	4	0.0013	[0.0000–0.9297]
	5	0.0452	[0.0000–0.9217]
	6	**0**.**6753**	[0.5934–0.9538]
	7	0.2739	[0.1064–0.9345]

In our analysis of *P*. *tomentosa* mb1, scenario 5 was again the most well supported model (PP = 0.9780; 95% confidence interval: 0.99972–1.0000; Table 2). This scenario suggests that *P*. *tomentosa* mb1 originated from the hybridization of *P*. *alba* and *P*. *adenopoda* (Fig. 5). Our analyses of *P*. *tomentosa* mb2 indicated that scenario 6 was the most likely (PP = 0.6753; 95% confidence interval: 0.5934–0.9538; Table 2). These results suggest that *P*. *davidiana* hybridized with *P*. *adenopoda* first, then hybridized with *P*. *alba* and generated *P*. *tomentosa* mb2 (Fig. 6).

## Discussion

Our results suggested that the genetic relationships among taxa in section *Leuce* were more complex than expected. Phylogenetic analyses show that *P*. *hopeiensis* clusters with *P*. *davidiana*, *P*. *alba* and some *P*. *tomentosa* in our nuclear DNA phylogeny and with *P*. *davidiana* in cpDNA phylogeny (Figs 1, S1 and S2). The change of phylogenetic location implies a hybrid origin^45^. *P*. *hopeiensis* is a mosaic of *P*. *alba* and *P*. *davidiana* at *K* = 2, but it is assigned to a unique cluster at *K* = 3 (Fig. 3B), which is strongly indicative of hybrid origin, moreover, this is emerging as a common pattern in many other hybrids^46^. DIYABC simulation analyses also suggest that *P*. *hopeiensis* is generated by hybridization between *P*. *davidiana* and *P*. *alba*. Indeed, it is believed that *P*. *hopeiensis* has obvious lineage of *P*. *davidiana* due to the morphological similarities between them, and it is likely formed by asexual propagation^47^. It is reasonable to believe that *P*. *davidiana* served as the female parent of *P*. *hopeiensis* since they are sister groups in the cpDNA phylogenetic tree (Fig. 1).

Our NewHybrids analysis suggests that almost all individuals of *P*. *hopeiensis* we sampled are F_1_s, only two are F_2_s, and no post F_2_ hybrids are found (Fig. 4A). The progeny of *P*. *hopeiensis*, obtained by crossing experiments, are morphologically variable, which seems to prove this point^47^. In fact, many hybridizations within genus *Populus* are also limited to F_1_s^48^. For instance, the great majority individuals of *P*. × *canescens* are F_1_ hybrids between *P*. *alba* and *P*. *tremula*^29^. Here, it seems plausible that advanced generations *P*. *hopeiensis* would occur naturally given the presence of some F_2_s and especially because we observe several large individuals of *P*. *hopeiensis* that are old enough to produce advanced generation hybrids. It is also surprising that no individual is identified as the product of the backcrossing of *P*. *hopeiensis* with either parent species. The absence of post-F_1_ hybrids may be resulted from hybrid sterility and F_2_ breakdown^49^.

Our analysis proves that *P*. *tomentosa* is also a hybrid. A concatenated nuclear phylogenetic tree was recovered with statistical phasing of alleles from original direct sequences. For any individual of *P*. *tomentosa*, one of its haplotype sequences is clustered with *P*. *adenopoda*, and the other is grouped with *P*. *hopeiensis* and *P*. *alba* in the nuclear phylogenetic tree (Fig. S2). This topology is the same as that of hybrid origin of *P*. *tomentosa*. Two distinct genetic types of *P*. *tomentosa* (mb1 and mb2) are identified in the cpDNA tree where mb1 clusters with *P*. *adenopoda*, and mb2 clusters with *P*. *davidiana* and *P*. *hopeiensis* (Fig. 1). Therefore, phylogenetic analyses imply *P*. *tomentosa* exhibits close affinity with *P*. *hopeiensis*, *P*. *alba*, *P*. *adenopoda* and *P*. *davidiana*. It is not surprising that *P*. *tomentosa* has a close relationship with *P*. *hopeiensis*, considering that *P*. *hopeiensis* is a hybrid of *P*. *alba* and *P*. *davidiana*. Clustering results of PCA also suggest that *P*. *tomentosa* is related to *P*. *adenopoda*, *P*. *alba* and *P*. *davidiana*, and has a closer affinity with the first two (Fig. 2). *P*. *tomentosa* could be split into two genetic types (mb1 and mb2) along the division recovered in the cpDNA phylogenies (Fig. 3A). *P*. *tomentosa* mb1 is a mixture of *P*. *adenopoda* and *P*. *alba* (Fig. 3A, *K* = 3; Fig. 3C, *K* = 2), and it is recovered as a distinct population isolated from *P*. *alba* and *P*. *adenopoda* at *K* = 3 (Fig. 3C). This result strongly suggests that *P*. *tomentosa* mb1 is a natural hybrid between *P*. *adenopoda* and *P*. *alba*. *P*. *adenopoda* is the female parent, because *P*. *tomentosa* mb1 first clusters with *P*. *adenopoda* in the cpDNA tree, and without doubt, *P*. *alba* is the paternal parent. DIYABC also provides further evidence for a hybrid origin of *P*. *tomentosa* mb1 (Table 2, Fig. 5). Although we detect a small amount of genetic material from *P*. *davidiana* in four individuals of *P*. *tomentosa* mb1 (Fig. 3A), we hypothesize that this material is contributed by admixed individuals of *P*. *adenopoda* through hybridization with *P*. *alba* to generate *P*. *tomentosa* mb1(discussed below).

STRUCTURE analysis indicates that the genetic material of *P*. *tomentosa* mb2 is a mosaic of various other groups of taxa (Fig. 3A): *P*. *alba*, *P*. *adenopoda*, and *P*. *davidiana*; *P*. *alba*, *P*. *davidiana*, and *P*. *tomentosa* mb1; or *P*. *alba*, *P*. *adenopoda*, and *P*. *hopeiensis*. Therefore, five taxa (*P*. *alba*, *P*. *adenopoda*, *P*. *davidiana*, *P*. *tomentosa* mb1, and *P*. *hopeiensis*) are potential parents of *P*. *tomentosa* mb2. *P*. *tomentosa* mb2 first clusters with *P*. *davidiana* in cpDNA phylogeny (Fig. 1), which indicates that *P*. *davidiana* is most likely to serve as the maternal parent in single cross or in either cross of trihybrid cross. Therefore, possible combinations (♀ × ♂) are *P*. *davidiana* × (*P*. *adenopoda* × P. *alba*) (same as *P*. *davidiana* × *P*. *tomentosa* mb1); (*P*. *davidiana* × *P*. *adenopoda*) × *P*. *alba*; (*P*. *davidiana* × *P*. *alba*) × *P*. *adenopoda* (same as *P*. *hopeiensis* × *P*. *adenopoda)*; and *P*. *davidiana* × (*P*. *alba* × *P*. *adenopoda*). DIYABC analysis suggest that trihybridization (*P*. *davidiana* × *P*. *adenopoda*) × *P*. *alba* is the most probable pattern (Table 2, Fig. 6). Subsequent hierarchical STRUCTURE analyses also support these three as potential parent species (Fig. 3A,D). Importantly, these are the parents proposed by Song^37^. Dickmann also speculated that *P*. *tomentosa* was a signal cross hybrid or a trihybrid and that its parents may have been either *P*. *alba* and *P*. *adenopoda* or *P*. *alba*, *P*. *adenopoda*, and *P*. *tremula* (note that, in this work, *P*. *davidiana* is referred to as a geographic variety of *P*. *tremula*)^50^. Although natural three-way hybrids have been reported, they are very rare to our knowledge^13,20^. For example, only a single trihybrid individual is detected across the three species *P*. *deltoides*, *P*. *nigra*, and *P*. *balsamifera*^13^. We identified 18 individuals as trihybrids, which represents 7.7% of all *P*. *tomentosa* specimens examined (Table 1). This rarity may be caused by the difficulty of trihybridization.

All *P*. *tomentosa* mb1 examined are F_1_ hybrids (Fig. 4B), explaining why *P*. *tomentosa* fertility is low. We predict that *P*. *tomentosa* had originated at least twice and in multiple regions because two types with different parents existed in different regions. *P*. *tomentosa* mb1 and mb2 with different origins and different genetic characteristics are likely to be perpetuated by asexual propagation^35^. In fact, asexual propagation is a very common in *Populus*^27,51^. Together, *P*. *tomentosa* mb1 and mb2 increase the diversity of *P*. *tomentosa* and have caused many debates about its origins.

Four specimens that we identify as *P*. *adenopoda* based on overall morphology (three from Hubei and one from Guizhou) exhibit several morphological similarities to *P*. *davidiana*, including oval leaves that are not glandular punctate. These individuals are clustered together with *P*. *davidiana* in nuclear phylogenetic tree and locate between *P*. *davidiana* and other *P*. *adenopoda* individuals in PCA analysis, suggesting their close relationship with *P*. *davidiana* (Figs 2 and S2). STRUCTURE analyses indicate that these individuals are a mixture of *P*. *davidiana*, which is similar to results that might be expected of a hybrid (Fig. 3A). We postulate that this genetic pattern is the result from introgression by *P*. *davidiana*. This hypothesis is strongly supported by the STRUCTURE analysis, which demonstrates that the admixed individuals of *P*. *adenopoda* display a mosaic cluster pattern at *K* = 2 and *K* = 3 (Fig. 3E). We would expect that a true hybrid would fall into a single cluster at higher *K* values (inferred from Gompert, *et al*.^46^). Single copy and neutral nuclear DNA markers have previously been used to refute alterative hypotheses, such as convergence and symplesiomorphy^51^. Indeed, introgression of varying degrees has been demonstrated in 82 genera of angiosperms, including *Populus* and *Salix*^9^. Finally, we observe this admixture in only a few specimens of *P*. *adenopoda*, indicating that gene flow between the species is restricted to a very small area. Heiser termed this pattern ‘localized introgression’^52^. This finding is consistent with the geographic scale of introgression, which is dependent on the geographical location of hybridization and the dispersal ability of hybridized offspring^11^. Species that are incompletely genetically isolated may exchange genes uni- or bidirectionally^53^. It is worth noting that the gene flow is unidirectional from *P*. *davidiana* to *P*. *adenopoda*. Such asymmetric gene flow has previously been demonstrated to be common in Salicaceae^25,54^. For example, asymmetric introgression has been detected from *P*. *fremontii* Wats. (section *Aigeiros*) to *P*. *angustifolia* James (section *Tacamahaca*)^55^. Although the causes of unidirectional gene flow remain unclear, contributing factors may include incongruent flowering times;^56^ species abundance effects within the hybrid zone^25,57^; species biases, where only those hybrids having a particular maternal species are viable^58^; epistatic interactions^13^; and heterogametic sex determination^59^. Our field investigations indicate that *P*. *adenopoda* and *P*. *davidiana* have similar flowering times (March to April) and are present in similar numbers in adjacent areas. In addition, both phylogeny and STURCTURE analyses indicate that either *P*. *adenopoda* or *P*. *davidiana* could act as female parents for *P*. *tomentosa*. Thus, epistatic interactions and heterogametic sex determination might be the key for unidirectional gene flow in poplar, but this hypothesis requires more testing.

## Materials and Methods

### Poplar taxa and individuals

We selected *P*. *hopeiensis*, *P*. *tomentosa*, *P*. *alba*, *P*. *adenopoda*, *P*. *davidiana*, *P*. *tremula*, *P*. *tremuloides*, and *P*. *grandidentata* as the objects of this study. We performed range-wide sampling of representative populations from 2010 to 2016 (392 individuals in 36 populations of 8 taxa, detailed sampling information is listed in Table S4, S5). Although triploids have been found in *P*. *tomentosa*, we did not collect them based on our previous records^60^. Conspecific specimens collected from the same geographic location were grouped as ‘populations’, for convenience, even though some ‘populations’ included only a few individuals. *P*. *lasiocarpa* Oliv. (section *Leucoides*) was selected as an outgroup based on previous work^33^. Fresh leaves of all selected trees were collected and stored in silica gel.

### DNA extraction, polymerase chain reaction (PCR), and sequencing

Total DNA was isolated from collected leaves with a Plant Genomic DNA Kit DP320 (Tiangen, Beijing, China). The integrity of all DNA was tested with 1% agarose gel electrophoresis.

We used six cpDNA primer pairs and ten single-copy nuclear DNA primers for PCR and sequencing (Table S6). Four of the cpDNA primers (*trn*k, *psbM-trnD*^*GTC*^, *rpoB-trnC*^*GCA*^, and *atpI-atpH*) were modified from Demesure, *et al*.^61^ and Shaw, *et al*.^62^; the remaining two (YLT9, YLT24) were from Wang, *et al*.^63^. Nine of the nuclear DNA primers were modified from Du, *et al*.^39^.

PCR amplifications were performed in a reaction volume of 30 μL, containing 3 μL 10 × PCR Buffer, 0.12 mM dNTPs, 0.75 U Taq DNA polymerase, 0.2 μM of each primer, and 30 ng of genomic DNA. PCR conditions were described by Wang *et al*.^63^. PCR products were sequenced directly with the amplification primers after purification with a TIANgel Midi Purification Kit (Tiangen, Beijing, China). Bidirectional sequencing was used if the length of sequences was greater than 800 bp. When a clear sequence was not obtained, PCR products were cloned with the pGEM-TEasy Vector System II (Promega, Madison, USA). Then, six to ten positive clones were randomly selected and sequenced with M13. Sequences generated were deposited in GenBank (accessions numbers MF512193-MF521199 and MG202418-MG203618). All the sequences were aligned and refined with Bioedit^64^. The phase program with default algorithm in software DnaSP v5 was used to phase the alleles of nuclear DNA, with the clone sequences of *P*. *tomentosa* as the known sequence^40,65^. In this process, degenerate bases of the original sequence will be diverged. The diverged haplotype sequences then were used for subsequent analyses.

### Neutral test and genetic diversity analyses

DnaSP v5^40^ was used to calculate nucleotide diversity (π)^66^, Watterson’s parameter (*θ*_w_)^67^, the number of segregating sites (S), and the number of haplotype (Nh) for each loci of all taxa.

To test the neutrality of variation, we used DnaDP v5^40^ to calculate Tajima’s *D*^68^ as well as Fu and Li’s D* and F*^69^. For loci that all indices calculated by DnaSP were significant, we used MLHKA^70^ to further judge whether they departed from neutrality. That is, the maximum likelihood (ML) ratio between the average ML value of a given locus in neutrality and the average ML value of them in nonneutrality was calculated with 3 independent runs of 100,000 sweeps each. Then, a chi-square test was performed (p < 0.05 was considered significant).

### Phylogenetic analyses

We used seven tests in RDP3 to assess for potential recombination events for nuclear DNA^71^. The nonrecombined fragments were trimmed and further analyzed. We used the simple indel coding method in GapCoder to code all DNA indels generated after alignment^72^. Jmodel test 2.1.4^73^ was used to decide the best nucleotide substitution model for all loci under the Akaike Information Criterion. Given cpDNA is maternally inherited and conserved, we combined all six cpDNA loci for phylogenetic analysis. Conversely, phylogenetic analyses of the combined nuclear DNA were performed under a partition scheme (ten data subsets: partitioned by ten loci) by using the models determined by Jmodeltest for of each locus.

PAUP* 4.0b10*^74^ was used to conduct a maximum parsimony (MP) analysis for cpDNA. An MP heuristic search performed with 1000 replicates of random taxon addition with tree bisection and reconnection (TBR) branch swapping, without steepest descent, and with unordered, equally weighted characters. To assess topological robustness, we performed 1000 bootstrap replicates with the same options. We used RAxML^75^ to analyze ML for cpDNA using two sets of 1000 rapid bootstrap replicates. ML was also performed employing IQ-TREE-1.6.6 using separate models for nuclear DNA with 1000 ultrafast bootstrap approximation (UFBoot)^76^. We also analyzed nuclear and chloroplast DNA with Bayesian inference in MrBayes v3.2.1^77^. Four independent MCMC analyses were run for 3,000,000 or 100,000,000 generations each with sampling every 1000 generations. We assumed that the dataset had reached convergence when the average standard deviation of split frequencies was less than 0.01. 25% of the samples were discarded as burn-in. All trees and edge support values were visualized in Figtree v1.4.0^78^.

### Genetic structure

To detect complex patterns of genetic structure, clustering analysis for nuclear DNA was conducted with principal component analysis (PCA) in GENALEX 6.5^41^. Phased nuclear DNA sequences were used for PCA analysis.

We first used STRUCTURE v2.3.3^42^ to assign the eight taxa to several different genetic clusters (*K*) based on ten nuclear DNA loci and determine the potential parents of hybrids. Then, three hierarchical STRUTURE analyses with only hybrids and their potential parents included were run again to further verify the previous inference. We also analyzed mechanisms of gene exchange between *P*. *adenopoda* and *P*. *davidiana* with STRUTURE separately. In this process, we divided *P*. *adenopoda* into two groups depending on whether it was introgressed by *P*. *davidiana*. An admixture model with correlated allele frequencies between populations was used. Considering the relationship between *P*. *adenopoda* and *P*. *davidiana* presented in the phylogenetic tree, we used the location information (usepopinfo = 1) to detect migrant individuals in the first structure analysis (including eight taxa) but not in the later hierarchical analyses. Moreover, we also run structure without location information (usepopinfo = 0) for eight taxa. We performed ten independent runs for each possible value of *K* from one to ten or five with a burn-in of 100,000 followed by 200,000 MCMC iterations. The most likely value of *K* based on the negative natural log likelihood of the data (*Ln*P(*K*)) and ∆*K*^ 79^ was calculated using STRUCTURE HARVESTER^80^. CLUMPAK^81^ was used to create and visualize population bar plots. To avoid cluster departure caused by differences in quantity among different populations, only haplotypes were used in analyses.

### Identification and classification of hybrids

We used Newhybrids v1.1^43^ to classify the genotype of hybrids and their parents based on posterior probability. For *P*. *hopeiensis* and its parent species, the six genotype classes used were pure parent *P*. *alba* (Phal), pure parent *P*. *davidiana* (Phda), F_1_ generation, F_2_ generation, backcross with *P*. *alba* (Bhal), and backcross with *P*. *davidiana* (Bhda). Only *P*. *tomentosa* mb1 and its parent species were analyzed given that Newhybrids v1.1 limits the allowed number of parents. The six genotype classes used for *P*. *tomentosa* were pure parent *P*. *alba* (Ptal), pure parent *P*. *adenopoda* (Ptad), F_1_ generation, F_2_ generation, backcross with *P*. *alba* (Btal), and backcross with *P*. *adenopoda* (Btad). We performed two replicate runs with a burn-in of 1,000,000 iterations followed by 1,500,000 sweeps in Jeffreys-like and uniform prior respectively. Default genotype categories were chosen. Together, the z and s options were also used to stipulate that some *P*. *alba* and *P*. *davidiana* or *P*. *adenopoda* and *P*. *alba* standard samples are of pure origin but preventing them from affecting the estimation.

### Approximate Bayesian computation

To derive a detailed origin pattern of *P*. *hopeiensis* and *P*. *tomentosa*, we compared plausible scenarios using approximate Bayesian computation (ABC) in DIYABC V 2.1.0^44^ using ten nuclear loci. Based on the results of our phylogenetic and STRUCTURE analyses, we only analyzed hybrids and their putative parental species with ABC.

We designed seven scenarios in three categories to explain the possible alternative origin pattern of *P*. *hopeiensis* and *P*. *tomentosa* mb1 (Fig. 5). Category 1 (scenario 1) modeled an ancestral split into three populations at time t0. Category 2 (scenarios 2, 3, and 4) modeled an ancestral split into two lineages at time t0 followed by a further diversification event in one of the lineages at time t1. Category 3 (scenarios 5, 6, and 7) was modeled as the split of two species at time t0 followed by hybridization and the generation of a new lineage at time t1.

Seven alternative scenarios were designed to explain the origins of *P*. *tomentosa* mb2 (Fig. 6): (1) *P*. *tomentosa* mb2 branched off from an ancestor directly; (2) *P*. *tomentosa* mb2 descended from *P*. *davidiana*; (3) *P*. *tomentosa* mb2 descended from the ancestor of *P*. *adenopoda* and *P*. *alba*; (4) *P*. *tomentosa* mb2 was the product of hybridization between *P*. *davidiana* and the ancestor of *P*. *adenopoda* and *P*. *alba*; (5) *P*. *adenopoda* hybridized with *P*. *alba* and then further hybridized with *P*. *davidiana* to generate *P*. *tomentosa* mb2; (6) *P*. *adenopoda* hybridized with *P*. *davidiana* and then further hybridized with *P*. *alba* to generate *P*. *tomentosa* mb2; and (7) *P*. *adenopoda* hybridized with *P*. *alba* and then further hybridized with *P*. *davidiana* to generate *P*. *tomentosa* mb2.

Historical models, genetic data and summary statistics parameters were listed in Table S7. To test these alternate theories, we ran 1,000,000 simulations for each scenario and selected the 1% of the simulated data closest to the observed data used to assess the posterior probabilities of all scenarios with logistic regression^44^.

## Supplementary information

Supporting information

## Data Availability

DNA sequences: Genbank accessions: MF512193-MF521199 and MG202418-MG203618.
