# Supplementary material for: Genetic analysis of admixture and hybrid patterns of Populus hopeiensis and P. tomentosa
Source: Sci Rep. 2019 Mar 18;9:4821. doi: 10.1038/s41598-019-41320-z (PMC6423230; doi:10.1038/s41598-019-41320-z)
Supplement: Supplementary file 1 — Supporting information [file 41598_2019_41320_MOESM1_ESM.pdf]

Genetic analysis of admixture and hybrid patterns of *Populus hopeiensis* and *P. tomentosa*

Dongsheng Wang [1,2], Zhaoshan Wang[1,3], Xiangyang Kang[4], Jianguo Zhang*[,1,3]

[1]State Key Laboratory of Tree Genetics and Breeding, Key Laboratory of Silviculture of the State Forestry Administration, Research Institute of Forestry, Chinese Academy of Forestry, Beijing 100091, China, [2]College of Horticulture Sciences & Technology, Hebei Normal University of Science & Technology, Qinhuangdao 066004, China, [3] Collaborative Innovation Center of Sustainable Forestry in Southern China, Nanjing Forestry University, 159 Nanjing 210037, Jiangsu Province, China, [4]College of Biological Sciences and Biotechnology, Beijing Forestry University, Beijing 100091, China

*Correspondence and requests for materials should be addressed to J.Z. (E-mail: zhangjg@caf.ac.cn)

D. W. and Z. W. contributed equally to this paper.

Table S1. The characteristics of the genes used in this study

Loci	Aligned length（bp）	Number of variable sites（%）	Number of informative sites （%）	GC (%)	Nucleotide substitution model
nDNA					
6	570	55（9.6）	41（7.2）	44.9	K80
12	425	33（7.8）	27（6.4）	55.1	TIM2
15	536	43（8.0）	39（7.3）	44.6	K80
18	529	43（8.1）	34（6.4）	39.7	K80
X12	488	26（5.3）	18（3.7）	45.7	TrNef
X14	799	59（7.4）	43（5.4）	42.5	TPM1uf
X15	685	61（8.9）	43（6.3）	42.6	HKY+I+G
X16	388	78（20.1）	55（14.2）	42.2	K80
X18	487	84（17.2）	60（12.3）	40.4	TrN
X19	808	76（9.4）	61（7.5）	38.5	TVMef
Combined data	5715	558（9.8）	421（7.4）	43.1	TIM3+G
cpDNA					
YLT9	1052	5（0.5）	5（0.5）	43.6	TPM2uf
YLT24	945	15（1.6）	11（1.2）	42.0	TPM1uf
trnK	2551	47（1.8）	31（1.2）	32.5	TVM+I
psbM-trnD^{GUC}	1176	19（1.6）	16（1.4）	29.8	TIM2+I
rpoB-trnC^{GCA}	996	16（1.6）	11（1.1）	30.0	TPM3uf+I
atpH-atpI	1021	15（1.5）	11（1.1）	27.8	TrN
Combined data	7741	117（1.5）	85（1.1）	34.3	TPM2uf+G

Table S2. Nucleotide diversity and neutral test of each locus

taxa	loci	S[①]	Θw[②]	π[③]	Tajima's D	Fu and Li's D*	Fu and Li's F*	Nh[④]	Hd[⑤]
P. ade	6	21	0.00888	0.01386	1.89541	1.34578	1.80238*	8	0.825
	12	1	0.00057	0.00103	1.19282	0.57433	0.86222	2	0.437
	15	7	0.00315	0.00447	1.19218	1.26229	1.45090	5	0.714
	18	11	0.00501	0.00558	0.35005	0.91256	0.86198	6	0.740
	X12	5	0.00247	0.00423	1.85517	1.12661	1.56957	2	0.413
	X14	19	0.00573	0.00561	-0.07144	-0.44629	-0.38144	8	0.790
	X15	14	0.00493	0.00682	1.23635	1.08930	1.33759	9	0.870
	X16	22	0.01367	0.01602	0.58513	1.67973*	1.55622	9	0.765
	X18	20	0.00992	0.01554	1.66506	1.34578	1.71275*	9	0.803
	X19	15	0.00448	0.00705	1.86564	1.55434*	1.94868*	8	0.684
	mean	14	0.00588	0.00802	1.17664	0.90130	1.03672	6.6	0.704
	YLT9	0	NA	NA	NA	NA	NA	1	0
	YLT24	0	NA	NA	NA	NA	NA	1	0
	trnK	3	0.00035	0.00021	-1.13127	-1.19315	-1.34945	3	0.307
	psbM-trnD^{GUC}	1	0.00028	0.00011	-1.16467	-1.49949	-1.61172	2	0.111
	rpoB-trnC^{GCA}	1	0.00032	0.00012	-1.16467	-1.49949	-1.61172	2	0.111
	atpH-atpI	0	NA	NA	NA	NA	NA	1	0
	mean	1	0.00032	0.00015	-1.15354	-1.39738	-1.52430	1.7	0.088
P. alb	6	12	0.00495	0.00693	1.23095	0.45552	0.83183	8	0.751
	12	3	0.00166	0.00190	0.31251	0.91572	0.85668	3	0.524
	15	5	0.00219	0.00324	1.21313	1.11690	1.33799	6	0.706
	18	15	0.00667	0.00991	1.55095	1.56041*	1.83679*	8	0.841
	X12	2	0.00096	0.00106	0.18661	-0.82800	-0.61833	3	0.499
	X14	11	0.00353	0.00419	0.57172	0.45552	0.58052	11	0.842
	X15	10	0.00343	0.00283	-0.52332	0.23920	-0.00550	9	0.685
	X16	12	0.00727	0.00450	-1.17361	-2.09350	-2.11372	9	0.737
	X18	8	0.00387	0.00297	-0.92678	0.73307	0.24191	8	0.792
	X19	16	0.00466	0.00525	0.40777	0.76293	0.76202	12	0.909
	mean	9	0.00392	0.00428	0.28500	0.19526	0.20816	7.7	0.729
	YLT9	0	NA	NA	NA	NA	NA	1	0
	YLT24	0	NA	NA	NA	NA	NA	1	0
	trnK	11	0.00125	0.00084	-1.16859	-1.64521	-1.74771	8	0.805
	psbM-trnD^{GUC}	1	0.00028	0.00039	0.72261	0.64952	0.76517	2	0.395
	rpoB-trnC^{GCA}	4	0.00126	0.00097	-0.65164	-0.75932	-0.84039	5	0.663
	atpH-atpI	0	NA	NA	NA	NA	NA	1	0
	mean	3	0.00093	0.00073	-0.36587	-0.58500	-0.60764	3	0.311
P. dav	6	15	0.00573	0.00379	-1.01768	0.15396	-0.29458	14	0.865
	12	14	0.00768	0.00615	-0.59976	-0.32008	-0.49312	15	0.858
	15	18	0.00731	0.00427	-1.28557	-0.83335	-1.17607	18	0.866
	18	8	0.00329	0.00298	-0.25102	-0.16554	-0.22775	8	0.480
	X12	9	0.00401	0.00275	-0.86355	-1.35457	-1.40611	9	0.595

	X14	26	0.00708	0.00369	-1.54827	-0.07404	-0.71612	21	0.89
	X15	21	0.00667	0.00403	-1.24874	-0.86523	-1.18880	22	0.914
	X16	37	0.02109	0.02049	-0.26089	-0.07977	-0.17445	36	0.977
	X18	47	0.02105	0.01611	-0.85033	-1.19594	-1.27497	21	0.916
	X19	30	0.00808	0.00485	-1.37781	-1.66218	-1.85775	23	0.934
	mean	23	0.00920	0.00691	-0.93036	-0.63967	-0.88097	18.7	0.830
	YLT9	3	0.00073	0.00081	0.25201	-0.24045	-0.11559	4	0.672
	YLT24	0	NA	NA	NA	NA	NA	1	0
	trnK	14	0.00154	0.00107	-1.02483	-1.15571	-1.30636	13	0.899
	psbM-trnD^{GUC}	5	0.00128	0.00136	0.17913	0.28709	0.29673	5	0.638
	rpoB-trnC^{GCA}	12	0.00345	0.00125	-2.10734	-2.20440	-2.54362	4	0.206
	atpH-atpI	4	0.00106	0.00068	-0.91947	-1.93398	-1.90181	3	0.5
	mean	6	0.00161	0.00103	-0.72410	-1.04949	-1.11413	5	0.486
P. gra	6	0	NA	NA	NA	NA	NA	1	0
	12	0	NA	NA	NA	NA	NA	1	0
	15	0	NA	NA	NA	NA	NA	1	0
	18	6	0.00342	0.00605	2.63471	1.27166	1.88243*	2	0.533
	X12	0	NA	NA	NA	NA	NA	1	0
	X14	2	0.00075	0.00134	1.97021	0.90708	1.35232	2	0.533
	X15	2	0.00088	0.00052	-1.03789	-0.50381	-0.73427	3	0.342
	X16	4	0.00311	0.00550	2.40809	1.14136	1.69429*	2	0.533
	X18	0	NA	NA	NA	NA	NA	1	0
	X19	1	0.00037	0.00066	1.52862	0.68829	1.02938	2	0.533
	mean	2	0.00171	0.00281	1.50075	0.70092	0.54914	1.6	0.247
	YLT9	0	NA	NA	NA	NA	NA	1	0
	YLT24	0	NA	NA	NA	NA	NA	1	0
	trnK	1	0.00015	0.00010	-1.05482	-1.12639	-1.20353	2	0.25
	psbM-trnD^{GUC}	0	NA	NA	NA	NA	NA	1	0
	rpoB-trnC^{GCA}	0	NA	NA	NA	NA	NA	1	0
	atpH-atpI	0	NA	NA	NA	NA	NA	1	0
	mean	1	0.00015	0.00010	-1.05482	-1.12639	-1.20353	1.2	0.042
P. hop	6	14	0.00461	0.00811	2.03500	0.96300	1.61663	7	0.587
	12	17	0.00751	0.00624	-0.46764	-1.42800	-1.27776	10	0.744
	15	14	0.00490	0.00589	0.54137	0.96300	0.96489	7	0.599
	18	8	0.00284	0.00520	1.97671	0.42455	1.13866	4	0.565
	X12	10	0.00385	0.00648	1.71106	-0.79709	0.10444	6	0.568
	X14	13	0.00305	0.00609	2.62953	0.89404	1.8166*	6	0.605
	X15	23	0.00658	0.00693	0.15793	-0.99540	-0.65714	16	0.691
	X16	19	0.00919	0.00754	-0.62910	-1.93865	-1.72387	7	0.569
	X18	19	0.00734	0.01399	2.55849*	0.28504	1.36861	7	0.620
	X19	7	0.00163	0.00374	2.97541*	1.18231	2.12367*	8	0.711
	mean	14	0.00515	0.00702	0.99436	-0.04472	0.19181	7.8	0.626
	YLT9	3	0.00062	0.00035	-0.86069	-0.45271	-0.67310	4	0.351
	YLT24	0	NA	NA	NA	NA	NA	1	0

	trnK	11	0.00104	0.00050	-1.50034	-2.44318*	-2.5120*	8	0.464
	psbM-trnD^{GUC}	6	0.00130	0.00066	-1.21583	-2.36444	-2.34493	5	0.448
	rpoB-trnC^{GCA}	0	NA	NA	NA	NA	NA	1	0
	atpH-atpI	2	0.00044	0.00028	-0.62033	-0.93379	-0.97698	3	0.272
	mean	4	0.00085	0.00045	-1.04930	-1.25031	-1.33167	3.7	0.256
P. tom	6	27	0.00730	0.00848	0.41949	-0.38893	-0.05068	8	0.592
	12	5	0.00175	0.00377	1.99677	0.9173	1.55199	4	0.532
	15	8	0.00222	0.00471	2.25034*	-0.77492	0.39193	4	0.510
	18	13	0.00365	0.00696	2.05095	-0.78139	0.37693	7	0.547
	X12	7	0.00213	0.00378	1.49022	1.07493	1.47465	4	0.543
	X14	15	0.00279	0.00513	1.95800	0.83812	1.54974	11	0.611
	X15	19	0.00412	0.00662	1.48168	0.49254	1.08526	10	0.604
	X16	23	0.00882	0.01110	0.65611	-0.83239	-0.25593	12	0.607
	X18	27	0.00918	0.01659	2.12340	0.21214	1.26258	19	0.755
	X19	17	0.00313	0.00881	4.36106*	0.96864	2.82091*	9	0.544
	mean	16	0.00451	0.00760	1.52208	0.17260	0.82072	8.8	0.585
	YLT9	1	0.00016	0.00014	-0.09507	0.44701	0.32753	2	0.150
	YLT24	7	0.00123	0.00066	-0.96786	0.16040	-0.28179	5	0.189
	trnK	7	0.00046	0.00029	-0.77274	-0.80724	-0.94965	4	0.158
	psbM-trnD^{GUC}	5	0.00083	0.00059	-0.54726	0.96805	0.53884	3	0.158
	rpoB-trnC^{GCA}	6	0.00111	0.00095	-0.27844	1.05232	0.70597	2	0.143
	atpH-atpI	4	0.00068	0.00045	-0.59541	-0.43733	-0.58231	3	0.151
	mean	5	0.00075	0.00051	-0.542797	0.230535	-0.04024	3.2	0.158
P. tro	6	10	0.00451	0.00169	-2.01127*	-2.81536*	-3.00287*	9	0.590
	12	11	0.00726	0.00710	-0.07283	0.54989	0.41951	10	0.865
	15	10	0.00527	0.00403	-0.77218	-1.01543	-1.10004	10	0.870
	18	9	0.00437	0.00342	-0.69063	0.22745	-0.05865	8	0.810
	X12	1	0.00053	0.00093	1.21263	0.60275	0.88534	2	0.452
	X14	10	0.00322	0.00261	-0.61050	-1.75995	-1.64734	9	0.656
	X15	8	0.00300	0.00120	-1.84801	-2.40727	-2.61092	7	0.537
	X16	21	0.01416	0.01164	-0.63134	-1.57703	-1.50156	12	0.836
	X18	16	0.00846	0.01191	1.40470	0.46177	0.88541	11	0.899
	X19	22	0.00731	0.00493	-1.16518	-0.23528	-0.62185	14	0.884
	mean	12	0.00581	0.00495	-0.35259	-0.57257	-0.59446	9.2	0.740
	YLT9	0	NA	NA	NA	NA	NA	1	0
	YLT24	6	0.00211	0.00103	-1.72892	-1.88169	-2.10179	4	0.495
	trnK	3	0.00038	0.00022	-1.27826	-1.03687	-1.25085	3	0.385
	psbM-trnD^{GUC}	3	0.00094	0.00084	-0.31543	0.01678	-0.07884	4	0.692
	rpoB-trnC^{GCA}	1	0.00035	0.00041	0.32440	0.71557	0.69871	2	0.363
	atpH-atpI	3	0.00134	0.00061	-1.67053	-2.09051	-2.25469	3	0.275
	mean	3	0.00100	0.00062	-0.93375	-0.85534	-0.99749	2.8	0.368
P. tru	6	16	0.00752	0.00566	-0.87789	-1.56636	-1.58547	11	0.888
	12	8	0.00504	0.00653	0.94808	0.14046	0.43887	7	0.685
	15	11	0.00550	0.00589	0.24576	-0.43327	-0.24898	13	0.938

18	14	0.00709	0.00478	-1.14007	-1.19156	-1.37205	9	0.772
X12	9	0.00494	0.00474	-0.13396	1.37323*	1.07977	8	0.659
X14	11	0.00363	0.00197	-1.57888	-2.77211*	-2.81493*	6	0.746
X15	9	0.00352	0.00231	-0.84843	-1.90302	-1.85152	8	0.859
X16	32	0.02209	0.01877	-0.66743	-1.43813	-1.40491	15	0.920
X18	28	0.01598	0.01579	-0.04454	0.22897	0.16918	13	0.935
X19	20	0.00663	0.00571	-0.50368	0.16877	-0.04270	16	0.953
mean	16	0.00820	0.00722	-0.46010	-0.74927	-0.53531	10.6	0.836
YLT9	1	0.00031	0.00016	-1.14053	-1.32974	-1.44334	2	0.167
YLT24	6	0.00210	0.00106	-1.89423*	-2.31814*	-2.50464*	3	0.318
trnK	2	0.00027	0.00023	-0.38175	-0.37372	-0.42375	3	0.439
psbM-trnD^{GUC}	2	0.00065	0.00046	-0.84971	-0.37372	-0.55559	3	0.439
rpoB-trnC^{GCA}	2	0.00074	0.00037	-1.45138	-1.72038	-1.86451	2	0.167
atpH-atpI	1	0.00034	0.00017	-1.14053	-1.32974	-1.44334	2	0.167
mean	2	0.00074	0.00041	-0.99278	-1.02546	-1.14611	2.5	0.283

Note: ①the number of segregating site; ②Theta (per site) from S; ③nucleotide diversity; ④Number of Haplotypes; ⑤Haplotype (gene) diversity; *indicates statistical significance; NA failed to compute due to insufficient variation

P. adenopoda (*P. ade*), *P. alba* (*P. alb*), *P. davidiana* (*P. dav*), *P. hopeiensis* (*P. hop*), *P. tomentosa* (*P. tom*), *P. grandidentata* (*P. gra*), *P. tremuloides* (*P. tro*), *P. tremula* (*P. tru*)

Table S3. The results of MLHKA tests

taxa	model	description	lnL	Comparison	LRS (df)①	② P value	K③
P. tremuloides	R	Netural(all K=1)	-61.5756	—	—	—	—
	S	Selection at 6	-57.2667	R VS S	8.6178	0.0033*	0.1936
P. tremula	Y	Netural(all K=1)	-21.2398	—	—	—	—
	Z	Selection at YLT24	-19.3346	Y VS Z	3.8103	0.0510	5.8107

Note: ①Likelihood Ratio Statistics; ②$P < 0.05$; ③The selection parameter of the gene which was selected in the model; *indicates statistical significance

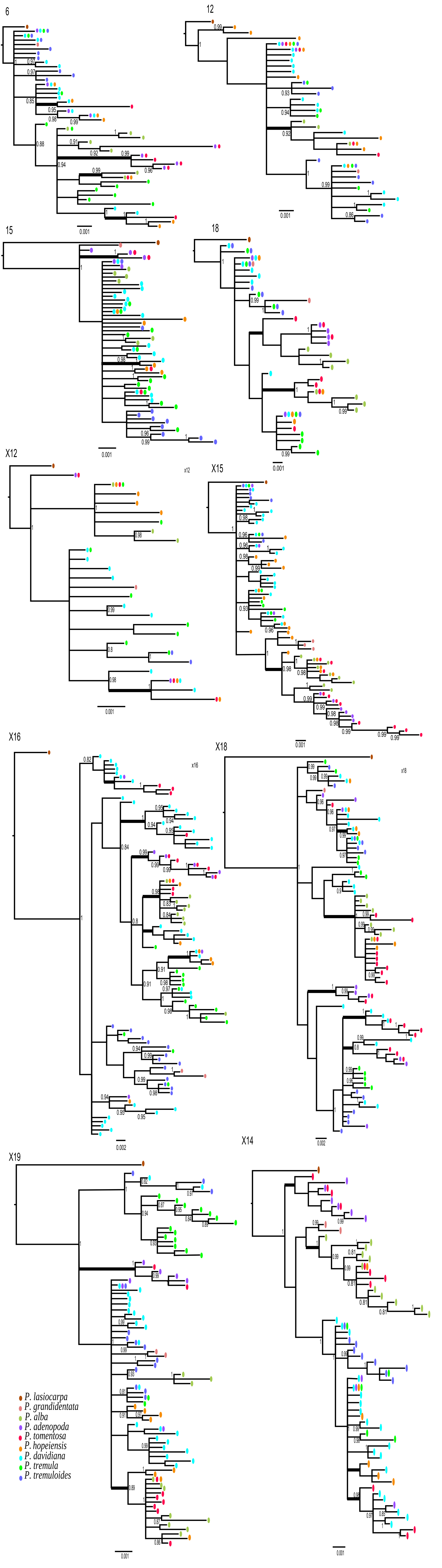

Figure S1. Bayesian inference (BI) phylogeny of section *Leuce* based on the individual nuclear DNA. Different colors indicate different haplotype sequences of taxa. Nodes less than 0.8 (posterior probability) are not represented. The branch length is proportional to the number of nucleotide substitutions measured with the scale bar. A thick black line indicates the clustering of *P. hopeiensis*, *P. alba* and *P. davidiana* or *P. tomentosa*. *P. adenopoda*, *P. davidiana* and *P. alba* with a high support.

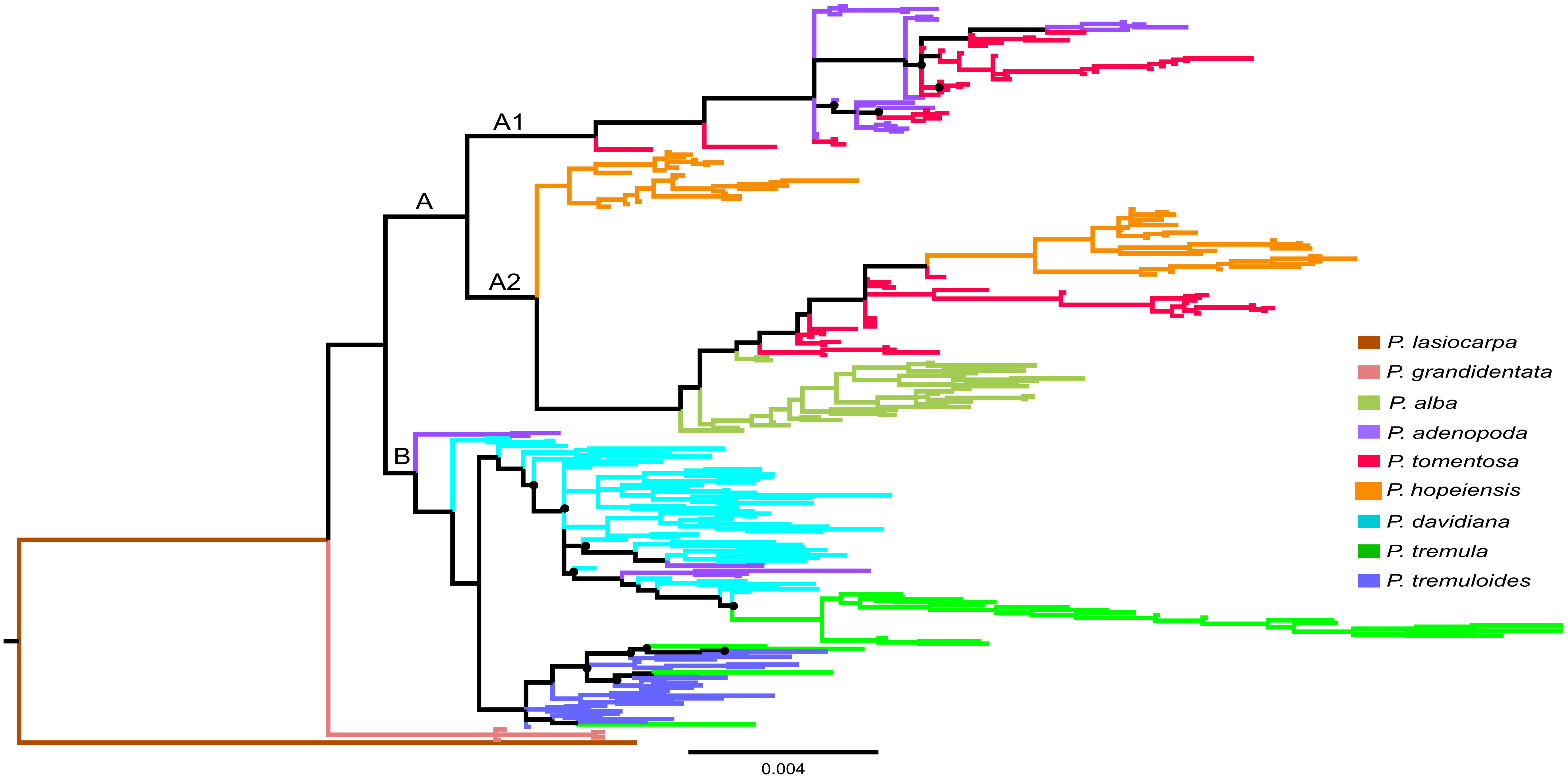

Figure S2. Bayesian inference (BI) phylogeny of section *Leuce* based on the combined ten nuclear DNA under the partition scheme. Different colors indicate different haplotype sequences of taxa. Nodes less than 0.9 (posterior probability) are represented by small black dots. The branch length is proportional to the number of nucleotide substitutions measured with the scale bar.

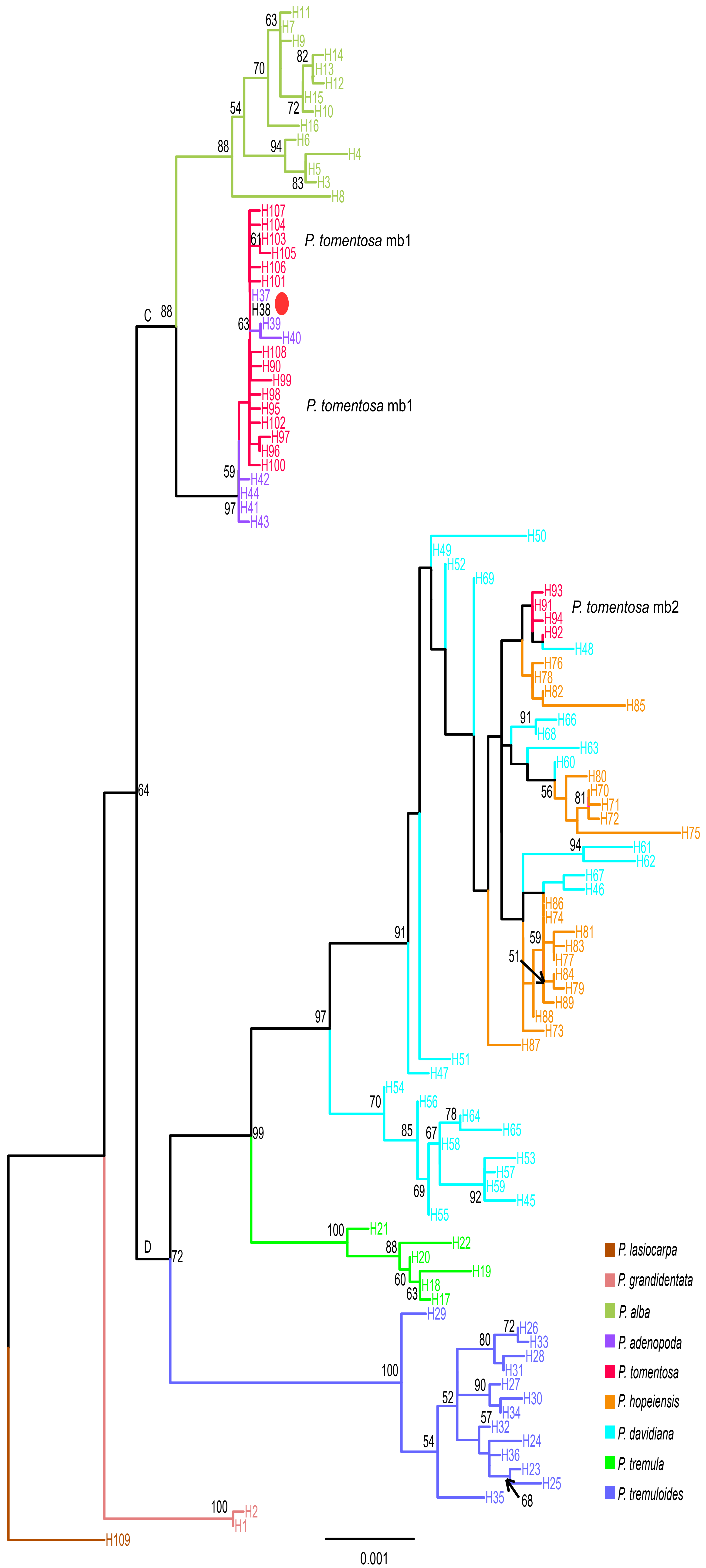

Figure S3. ML tree of section *Leuce* generated from 6 concatenated cpDNA regions with TPM2uf+G model. The numbers near the branches is the BS values of ML. Only these values that greater than 50 were shown. The branch length is proportional to the number of nucleotide substitutions measured with the scale bar. Different colors represent different taxa. Hx represents haplotypes number. The pie chart represents that Hap38 contains *P. adenopoda* (1 individual) and *P. tomentosa* (191 individuals).

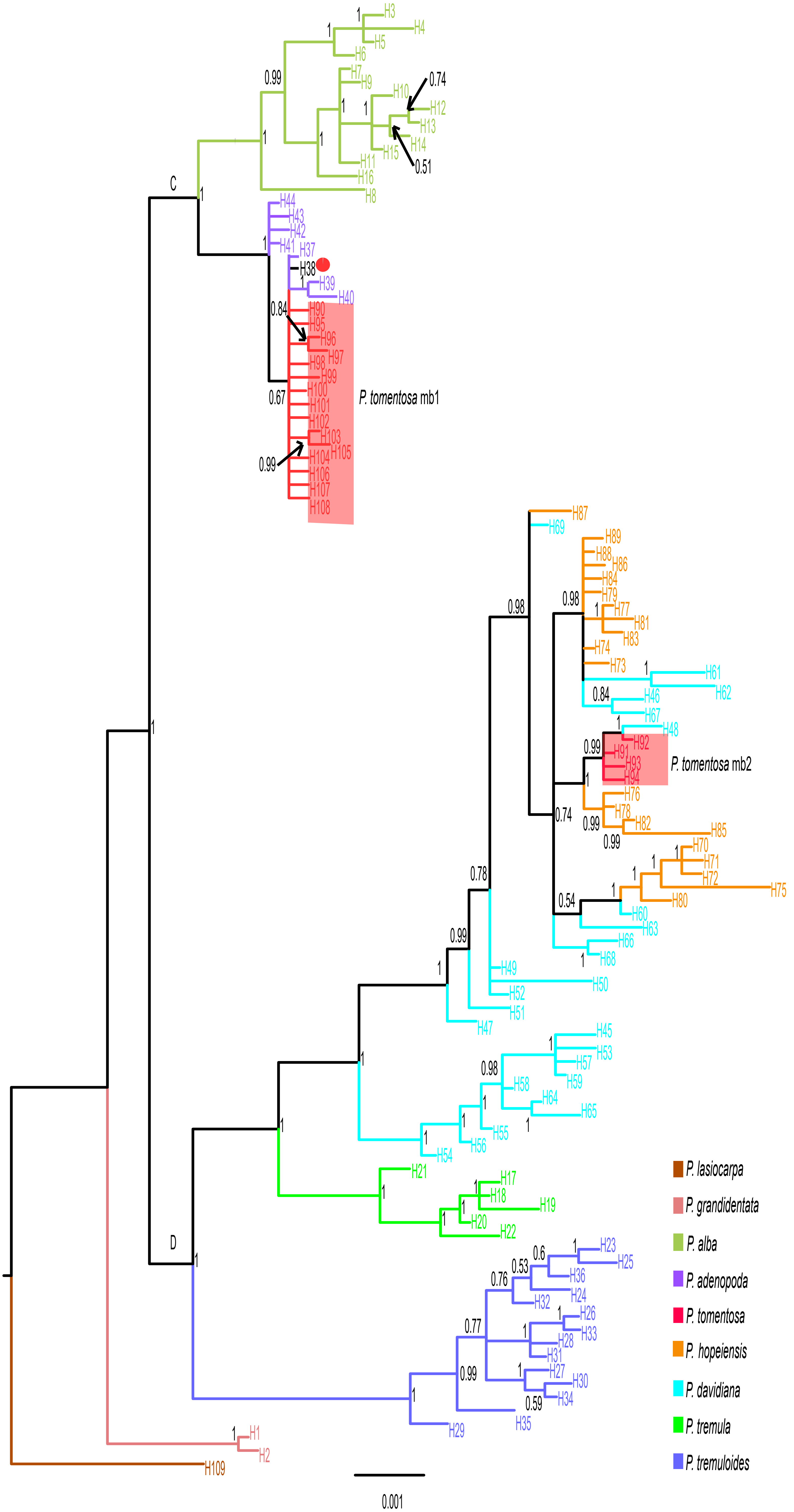

Figure S4. BI tree of section *Leuce* generated from 6 concatenated cpDNA regions with the TPM2uf+G model. The numbers near the branches is the PP values of BI. Only these values that greater than 0.50 were shown. The branch length is proportional to the number of nucleotide substitutions measured with the scale bar. Different colors represent different taxa. Hx represents haplotypes number. The pie chart represents that Hap38 contains *P. adenopoda* (1 individual) and *P. tomentosa* (191 individuals).

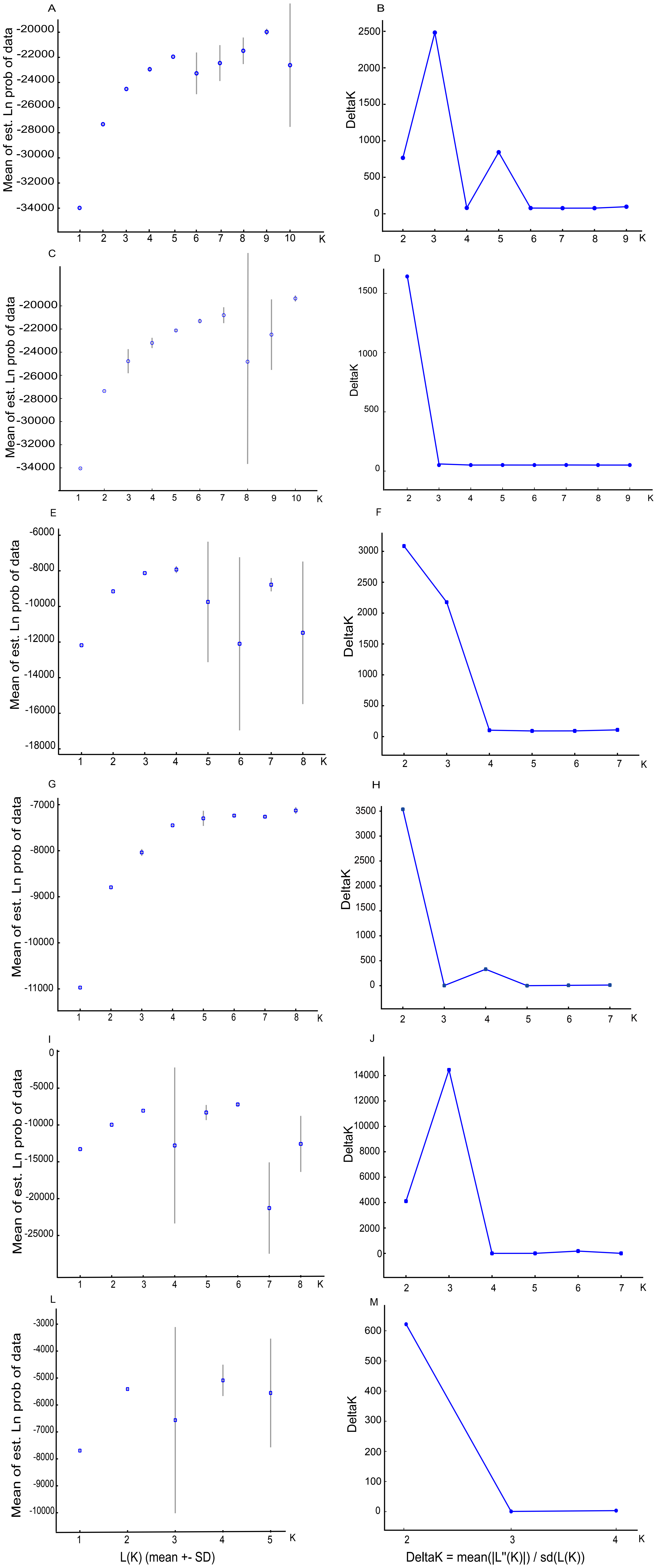

Figure S5. Log-likelihood values (ACEGI) and Delta K (BDFHJ) of STRUCTUR based on 10 nuclear DNA regions. AB for all taxa analyzed in this study, CD for *P. hopeiensis* and its putative parents, EF for *P. tomentosa* mb1 and its putative parents, GH for *P. tomentosa* mb2 and its putative parents, IJ for 'unmixed' *P. adenopoda*, 'admixed' *P. adenopoda* and *P. davidiana*.

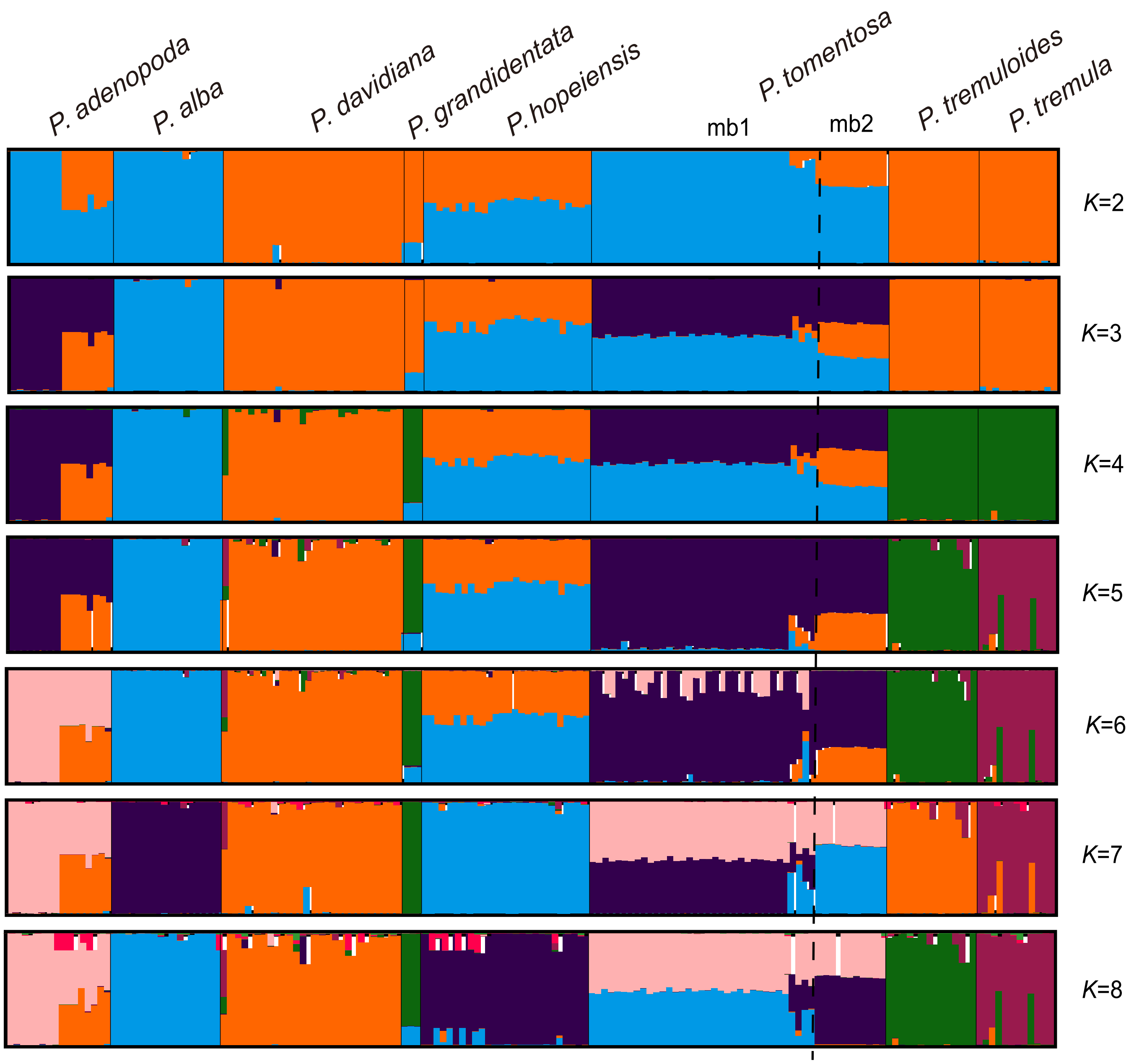

Figure S6. STRUCTURE results based on ten nuclear DNA dataset with usepopinfo=0. Taxa and subgroups are shown along the x-axis, and values of K are shown on the y-axis.

Table S4. Taxa of section *Leuce* and outgroup sampled in this study

Taxa	Code	Origin	No.	Taxa	Code	origin	No.
P. alba L.	YXJ	Xinjiang China	16	*P. davidiana* Dode	HLJ	Heilongjiang China	8
	YBL	Berlin Germany	4		QH	Qinhai China	2
			20		ZHBJ	Hebei China	2
P. adenopoda Maxim.	CQ	Chongqing China	4		ZGZ	Guizhou China	4
	XHBE	Hubei China	10		ZHN	Henan Chin	4
	XGZ	Guizhou China	4		ZGS	Gansu China	2
			18		XZ	Tibet China	2
P. tomentosa Carr.	MBJ*	Beijing China	36		ZBJ	Beijing China	2
	MHBJ*	Hebei China	53		NX	Ningxia China	2
	MHN*	Henan China	55				28
	SXJ*	Shanxi China	43	*P. tremula* L.	OBL	Berlin Germany	3
	MSXQ*	Shaanxi China	35		OXJ	Xinjiang China	4
	MGS*	Gansu China	6		ASL	Olso Noeway	1
	AH*	Anhui China	6		SLS	Zurich Switzerland	1
			234		OHXJ	Helsinki Feiland	3
P. tremuloides Michaux	QBK	Quebec Canada	1				12
	ABT	Alberta Canada	6	*P. hopeiensis* Hu & H. F. Chow	HGS	Gansu China	42
	WTH	Ottawa Canada	1		HSXQ	Shanxi China	15
	MVLY	Victoria Canada	4		HBJ	Beijing China	1
	MHXJ※	North America	2				58
			14	*P. lasiocarpa* Oliv.	DHBE	Hubei China	1
P. grandidentata Michx.	DVLY	Ottawa Canada	8	Total			393

Note: We selected specimens of *P. davidiana*, *P. tremula* and *P. tremuloides* randomly based on the research did by Du, et al.[1] to ensure the most haplotypes diversity and economy, so same population had only a few individuals. In order to convenience narration, specimens with the same origin grouped together and were named as a 'population' in this study, even if the 'populations' only comprised a few individuals.

*indicates: The most samples of *P. tomentosa* were collected from the germplasm repository of Guanxian in China, due to the lack of natural population. All *P. tomentosa* in the repository are elite trees by screening, and have never been crossbreeding. The detailed sources of *P. tomentosa* are shown in Table S5.

※indicates: MHXJ of *P. tremuloides* was collected from the germplasm repository of Punkaharju research center in

Helsinki Feiland.

Table S5. The detailed sources of *P. tomentosa*

Code	Origin	No.	Code	Origin	No.
MHBJ*(Hebei)	Haigang	2	SXJ*(Shanxi)	Taigu	1
	Funing	2		Yuncheng	1
	Changli	3		Xinjiang	1
	Lulong	1		Linyi	1
	Huailai	1		Jishan	2
	Xushui	3		Xiaxian	3
	Lixian	1		Yuanqu	4
	Anguo	2		Wenxi	2
	Dingzhou	1		Hejin	2
	Qingyuan	1		Pinglu	1
	Fuping	1		Fenxi	2
	Zhengding	2		Fushan	1
	Gaocheng	5		Puxian	1
	Yuanshi	2		Xiangfen	2
	Zanhuang	1		Linfen	1
	Zhaoxian	1		Wuxiang	1
	Jingjin	1		Zezhou	1
	Ningjin	1		Changzhi	1
	Shahe	1		Huguan	2
	Nangong	1		Luzhou	3
	Guangzong	1		Pingshun	1
	Weixian	2		Pingding	1
	Yongnian	1		Xiyang	2
	Daming	2		Mengxian	1
	Shexian	1		Zuoquan	2
	Linzhang	1		Qixian	1
	Qiuxian	1		Datong	2
	Langfang	2			43
	Sanhe	1	MSXQ*(Shaanxi)	Xian	25
	Bazhou	2		Baoji	1
	Xianxian	1		Fengxiang	1
	Cangxian	2		Fufeng	1
	Shenzhou	2		Yangling	2
	Jizhou	1		Zhouzhi	1
		53		Lantian	1
MHN*(Henan)	Xixian	2		Fuping	3
	Fanxian	1			35
	Weihui	1	MGS*(Gansu)	Tianshui	1
	Yanjin	1		Liangdang	2
	Xiuwu	1		Xihe	2

	Boai	1		Wenxian	1
	Weishi	1			6
	Wenxian	2	AH*(Anhui)	Huaiyin	5
	Qinyan	3		Yancheng	1
	Mengzhou	2			6
	Jiaozuo	2	MBJ*(Beijing)	Tongxian	2
	Shangqiu	6		Changping	6
	Kaifeng	6		Daxing	3
	Zhengzhou	7		Fangshan	2
	Mengjin	1		Miyun	2
	Xinan	1		Haidian	21
	Ruyang	1			36
	Luoyang	3			
	Taikang	2			
	Zhoukou	2			
	Changge	1			
	Linyin	1			
	Xuchang	2			
	Xiping	1			
	Zhumadian	3			
	Xianyang	1			
		55			

Table S6. Primers information

Loci	Primer sequences(5'-3')	Reference
Chloroplast DNA		
*trn*K	F: TCAGTGCTGGTTATCCAATTACAG	modified from Demesure, et al.[2]
	R: ATTGGATTTGCTGTGATA	
psbM-trnD^{GUC}	F: AGCAATAAATGCGAGAATATTTACTTCCAT	modified from Shaw, et al.[3]
	R: TACATCAATTCATCTCTTCAT	
rpoB-trnC^{GCA}	F: CTACAAAATCCTTCAAATTG	modified from Shaw, et al.[3]
	R: ATCTTGAATCCCATTTTTCTTAAT	
atpH-atpI	F: CCAACCCAGCAGCAATAAC	modified from Shaw, et al.[4]
	R: TATTTACAAGTGGTATTCAAGCT	
YLT9	F: ATACTTCGATTCTGCCCTTCG	Wang et al.[5]
	R: GGTTTGCCTTGGTATCGTGTT	
YLT24	F: TCGAGCCGACGAGATTAG	Wang et al.[5]
	R: TCTGGAGACCGAAATACCC	
Nuclear DNA		
6	F: TCTGCTTTCCACTTCTTGC	modified from Du et al.[1]
	R: CATACTCTCCCATTGTCCC	
12	F: TGGCAGAATCACCAGACCCTC	modified from Du et al.[1]
	R: CCAATTTAGCATCTTCAGCCTCAT	

15	F: GCCTCCTGATTATTATGC	modified from Du et al.[1]
	R: TATTACAAGCCCTTCCAG	
18	F: GTTTGTTGTTCTGTTGATTGT	modified from Du et al.[1]
	R: GGCTTCTCTTCTCTGATATTT	
X12	F: CACCACATCCCGCTTTCTCTCTTCACTT	modified from Du et al.[1]
	R: TAAACCCCAGGAGGCAAAACAGCACCAG	
X14	F: TGTTTGATGGACCTGGCTGCT	modified from Du et al.[1]
	R: CGGTTTATTGCCTTGTGGAGA	
X15	F: CTGAAAGGGAAAATAGTGGACAGTCAA	modified from Du et al.[1]
	R: GGATAACAGTAGCATGGAGATATGGAT	
X16	F: GGTAGCGTATTCAAAGATGGCAGAGG	
	R: TGGACGGACCAAGAAAAACGGAGGAT	
X18	F: TATGGAAAAAGTTATGCCAAGAGGA	modified from Du et al.[1]
	R: CAAAGGAGCAGAAGGCTATATCAAG	
X19	F: AAGTCTGGTCAAGGCAGTGGTC	modified from Du et al.[1]
	R: TCTGTGCTGTGATGTTTGGGGG	

Table S7. Prior distributions of the parameters used in DIYABC

Parameters		Taxa		
		P. hopeiensis	*P. tomentosa* mb1	*P.tomentosa* mb2
Sex ratio		1:1	1:1	1:1
Effective population size	N1	100-600000	100-800000	100-800000
	N2	100-1500000	100-1500000	100-600000
	N3	100-500000	100-1000000	100-3000000
	Na	100-1500000	100-1500000	100-3000000
	N4			100-150000
	N5			100-600000
Time scale in generations	t1	100-200000	100-300000	100-600000
	t2	100-600000	100-700000	100-400000
	t3			100-300000
	to			100-800000
Admixture rate	ra	0.001-0.999	0.001-0.999	
	r1			0.001-0.999
	r2			0.001-0.999
Mean mutation rate	μ	10^{-9}-10^{-8}	10^{-9}-10^{-8}	10^{-9}-10^{-8}
Mutation model	Kimura 2 Parameters			
One sample summary statistics	number of haplotypes, number of segregating sites, mean of pairwise differences, Tajima's D, variance of pairwise differences, private segregating sites			
Two sample summary statistics	number of haplotypes, number of segregating sites, mean of pairwise differences (W), mean of pairwise differences (B)			

References:

1 Du, S. *et al.* Multilocus analysis of nucleotide variation and speciation in three closely related Populus (Salicaceae) species. *Molecular ecology* **24**, 4994-5005**,** doi:10.1111/mec.13368 (2015).

2 Demesure, B., Sodzi, N. & Petit, R. J. A set of universal primers for amplification of polymorphic non‐coding regions of mitochondrial and chloroplast DNA in plants. *Molecular Ecology* **4**, 129-134**,** doi:10.1111/j.1365-294X.1995.tb00201.x (1995).

3 Shaw, J. *et al.* The tortoise and the hare II: relative utility of 21 noncoding chloroplast DNA sequences for phylogenetic analysis. *American Journal of Botany* **92**, 142-166 (2005).

4 Shaw, J., Lickey, E. B., Schilling, E. E. & Small, R. L. Comparison of whole chloroplast genome sequences to choose noncoding regions for phylogenetic studies in angiosperms: the tortoise and the hare III. *American Journal of Botany* **94**, 275-288**,** doi:10.3732/ajb.94.3.275 (2007).

5 Wang, D., Wang, Z., Du, S. & Zhang, J. Phylogeny of section Leuce (Populus, Salicaceae) inferred from 34 chloroplast DNA fragments. *Biochemical Systematics and Ecology* **63**, 212-217**,** doi:10.1016/j.bse.2015.09.020 (2015).
